# Leveraging open dataset and transfer learning for accurate recognition of chronic pulmonary embolism from CT angiogram maximum intensity projection images

**DOI:** 10.1186/s41747-023-00346-9

**Published:** 2023-06-21

**Authors:** Tuomas Vainio, Teemu Mäkelä, Anssi Arkko, Sauli Savolainen, Marko Kangasniemi

**Affiliations:** 1 Radiology, HUS Medical Imaging Center, University of Helsinki and Helsinki University Hospital, 00290 Helsinki, Finland; 2 Department of Physics, University of Helsinki, Helsinki, Finland

**Keywords:** Artificial intelligence, Computed tomography angiography, Deep learning, Neural networks (computer), Pulmonary embolism

## Abstract

**Background:**

Early diagnosis of the potentially fatal but curable chronic pulmonary embolism (CPE) is challenging. We have developed and investigated a novel convolutional neural network (CNN) model to recognise CPE from CT pulmonary angiograms (CTPA) based on the general vascular morphology in two-dimensional (2D) maximum intensity projection images.

**Methods:**

A CNN model was trained on a curated subset of a public pulmonary embolism CT dataset (RSPECT) with 755 CTPA studies, including patient-level labels of CPE, acute pulmonary embolism (APE), or no pulmonary embolism. CPE patients with right-to-left-ventricular ratio (RV/LV) < 1 and APE patients with *RV/LV* ≥ 1 were excluded from the training. Additional CNN model selection and testing were done on local data with 78 patients without the RV/LV-based exclusion. We calculated area under the receiver operating characteristic curves (AUC) and balanced accuracies to evaluate the CNN performance.

**Results:**

We achieved a very high CPE *versus* no-CPE classification AUC 0.94 and balanced accuracy 0.89 on the local dataset using an ensemble model and considering CPE to be present in either one or both lungs.

**Conclusions:**

We propose a novel CNN model with excellent predictive accuracy to differentiate chronic pulmonary embolism with *RV/LV* ≥ 1 from acute pulmonary embolism and non-embolic cases from 2D maximum intensity projection reconstructions of CTPA.

**Relevance statement:**

A DL CNN model identifies chronic pulmonary embolism from CTA with an excellent predictive accuracy.

**Key points:**

• Automatic recognition of CPE from computed tomography pulmonary angiography was developed.

• Deep learning was applied on two-dimensional maximum intensity projection images.

• A large public dataset was used for training the deep learning model.

• The proposed model showed an excellent predictive accuracy.

**Graphical Abstract:**

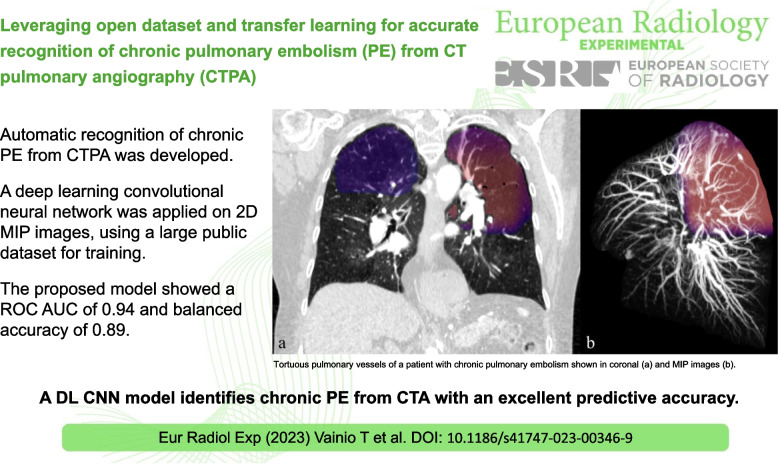

**Supplementary Information:**

The online version contains supplementary material available at 10.1186/s41747-023-00346-9.

## Background

Chronic pulmonary thromboembolism (CPE) is the only cause of pulmonary hypertension potentially curable by pulmonary endarterectomy, but the prognosis is poor if left untreated [1–3]. Additionally, longer delays in chronic thromboembolic pulmonary hypertension (CTEPH) diagnosis are associated with a higher risk of all-cause mortality [4]. However, early diagnosis is challenging, and the radiologic signs of CTEPH are often missed in computed tomography pulmonary angiography (CTPA) [5]. An automated classification tool could aid the radiologist in early detection and improve patient selection in clinical practice.

The CPE diagnosis is not based solely on thrombus or hypoperfusion detection but on many different radiological signs, including calibre variation and abrupt narrowing of pulmonary vessels, narrowing of distal vessels, and proximal artery tortuosity and dilatation [6–10].

Hence, we aimed to develop an alternative to our previous machine learning model on hypoperfusion detection [11] and evaluate its feasibility in classifying CPE from CTPA based on the overall morphology of pulmonary vasculature instead of manually handcrafting vascular features into robust descriptors. Automatic lung segmentation and two-dimensional (2D) maximum intensity projection (MIP) images from various angles of the CTPA were used as model inputs and the CPE diagnoses as the training and testing targets. In addition, we investigated which regions in the CTPA could be associated with the classification decision. This is the first study recognising chronic pulmonary embolism from 2D MIP images of a CTPA by a deep learning method.

## Methods

### Public dataset

For the initial training and selection of the convolutional neural network (CNN) model, we utilised the RSNA-STR Pulmonary Embolism CT (RSPECT) dataset from the Radiological Society of North America (RSNA), originally consisting of 12,195 CTPA studies, of which a subset was released for RSNA Pulmonary Embolism Detection AI Challenge 2020 [12]. Among other labels, diagnosis of acute or chronic PE was made publicly available for 7,279 CTPA studies. The available data was further rectified to unify the final training data by minimising the number of possible confounding factors and improving the class balance.

**Fig. 1:**
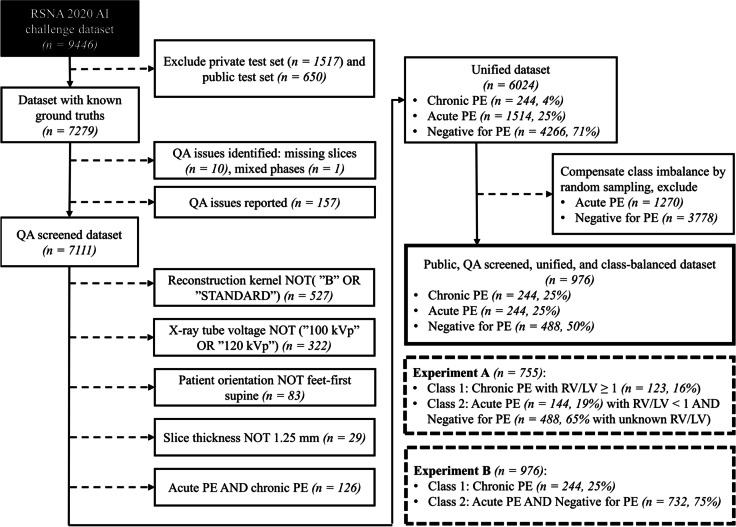
A flow chart of the data selection process for the public dataset. Dashed arrows indicate exam exclusions without overlap with previous exclusions. *PE*, Pulmonary embolism; *RV/LV*, Right ventricular to left ventricular diameter ratio; *QA*, Quality assurance

The exclusion process is described in Fig. 1, and full details of the RSPECT dataset are available [12]. We performed two initial experiments, hereafter referred to as experiments A and B, on the RSPECT data and chose the best-performing approach for ensemble model training and final testing on a local data set.

The data in experiment A consisted of training a neural network classifier aiming to distinguish patients with chronic PE and having a right-to-left-ventricular ratio (RV/LV) ≥ 1 (positive class) from a negative class comprised of both negative exams for PE and patients reported with acute PE with RV/LV < 1. We hypothesised that patients in the former category would demonstrate more features of CPE in their vascular morphology due to more advanced disease and elevated pulmonary hypertension. The RV/LV criterion on the latter category was applied to minimise the possibility of pulmonary hypertension and underlying misdiagnosed CPE in the control groups. The RV/LV ratio was unavailable for the negative for PE exams and was not used as an exclusion criterion. In experiment B, we retrained the same classifier without the RV/LV criteria.

### Local dataset

The local dataset was constructed from the hospital district picture archiving and information system by retrospectively reviewing reports of ventilation-perfusion (V/Q) scans performed between January 2017 and December 2019 and CTPA studies between July 2019 and October 2019 in Helsinki University Hospital. Based on the reports, we initially selected 30 patients with findings suggestive of CPE and 32 patients with no signs of pulmonary embolism in the V/Q scan for the study. Additionally, 34 patients with acute pulmonary embolism in the CTPA were initially selected.

The inclusion criteria for the positive CPE cases were a positive V/Q scan for CPE and a CTPA with signs of CPE performed in our hospital district within 3 months before or after the positive V/Q scan without signs of acute pulmonary embolism before treatment. The negative patients’ inclusion criteria were a negative V/Q scan for acute or chronic pulmonary embolism and a negative CTPA for acute or chronic pulmonary embolism performed within 3 months of the negative V/Q scan. The inclusion criteria for the patients with acute pulmonary embolism were a positive CTPA study for acute pulmonary embolism without signs of CPE and no CPE diagnosis or death within 2 years after the initial CTPA for APE to exclude possibly misdiagnosed CPE. CTPA studies with radiological signs of a parenchymal disease unrelated to CPE (*e.g.*, hyper-attenuation caused by talcosis) extending over two-thirds of the lung volume were excluded. In addition, artefacts caused by foreign material covering more than one-third of the lung volume in the CTPA were a criterion for exclusion.

After the exclusion, CTPA studies of 26 CPE, 26 negative, and 26 APE cases were included for CNN validation and testing. Patient characteristics are presented in Table 1. The acquisition and contrast media protocol were defined by the joint municipal authority for specialised healthcare but might have varied depending on the patient’s age, size, and renal function. Different tube voltages were used depending on the scanner and the size of the patient (Table 2).

**Table 1:** Local dataset patient characteristics

Patient group	Median age (min–max)	Female	Male	CTEPH	CTED	RV/LV ≥ 1	RV/LV < 1
CPE	66 (21–82)	17	9	19	7	24	2
Negative for PE	67 (33–88)	14	12	0	0	16	10
APE	63 (32–90)	16	10	0	0	7	19
All	65 (21–90)	47	31	19	7	47	31

*APE* Acute pulmonary embolism, *CPE* Chronic pulmonary embolism, *CTED* Chronic thromboembolic disease, *CTEPH* Chronic thromboembolic pulmonary hypertension, *RV/LV* Right ventricular to left ventricular diameter ratio, *PE* Pulmonary embolism

**Table 2:** Number of patients imaged with each computed tomography scanner model and tube voltage. The patients were imaged in nine different hospitals

Scanner model (manufacturer)	X-ray tube voltage				Total
	80 kVp	100 kVp	120 kVp	140 kVp	
Aquilon Prime (Toshiba)	0	1	0	0	1
Discovery HD (General Electric Healthcare)	0	4	0	0	4
LightSpeed VCT (General Electric Healthcare)	0	9	1	0	10
Revolution EVO (General Electric Healthcare)	0	6	2	0	8
Revolution HD (General Electric Healthcare)	1	5	0	1	7
SOMATOM Definition AS (Siemens Healthineers)	0	2	2	0	4
SOMATOM Definition Edge (Siemens Healthineers)	3	25	5	0	33
SOMATOM Definition Flash (Siemens Healthineers)	0	9	2	0	11
Total	4	61	12	1	78

### Dataset splits

**Fig. 2:**
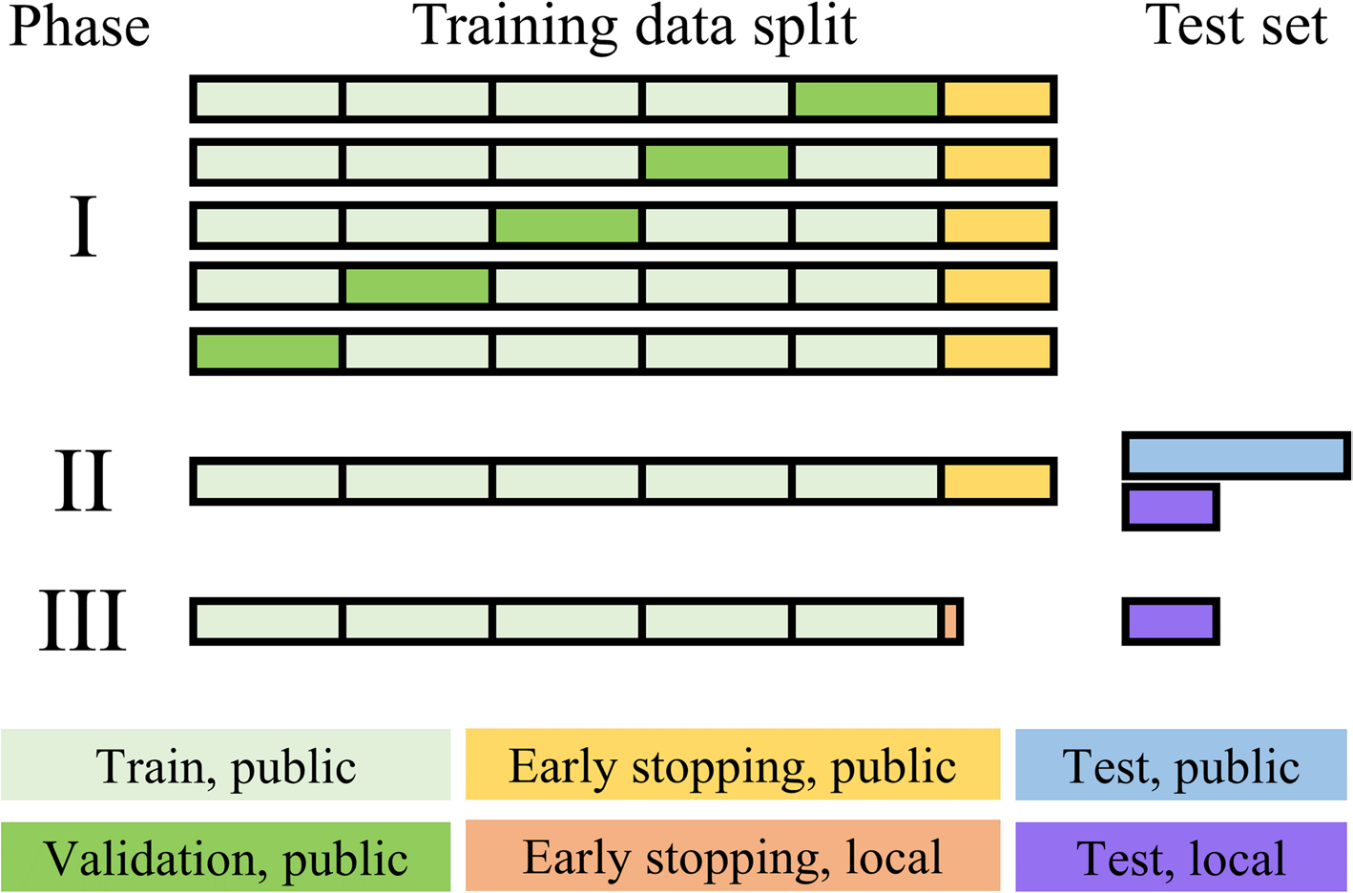
Data splits in phases 1–3. The block widths correspond approximately to the relative number of cases in the experiment A and the local sets. These test sets are not to be confused with the private and public test sets in the original RSPECT dataset (see text for details), for which we did not have ground truths available. Experiment A sets or splits were subsets of the corresponding sets or splits in experiment B

The CNN training and testing were divided into three phases with the following data splits (Fig. 2). Test sets were separated from the training set, with the patient’s left and right lungs always belonging to the same set. Phase 1 consisted of hyperparameter optimisation and model selection using fivefold cross-validation with separate sets for selecting the best-performing model checkpoint (hereafter referred to as “early stopping sets” to distinguish from the validation sets in the cross-validation and because they are analogous to stopping the training to prevent overfitting). In experiment A, the training set consisted of 86 CPE, 97 APE, and 338 negative-for-PE exams (multiply by two to count left and right lung volumes separately), the early stopping set of 14, 16, and 50 exams, and the test set of 23, 31, and 100 exams, respectively. In experiment B, the training set consisted of 169 CPE, 169 APE, and 338 negative exams for PE, the early stopping set of 25, 25, and 50 exams, and the test set of 50, 50, and 100 exams, respectively. In experiment B, the cross-validation performance for both A and B validation sets was recorded.

In phase 2, the best-performing network was retrained on the entire experiment A training and early stopping sets, *i.e.*, without cross-validation and using the same cases for model selection. We tested this model against experiment A and B test sets and the local test set consisting of 21 CPE, 21 APE, and 21 negative exams for PE. In phase 3, we utilised a tiny “local early stopping set” (5 CPE, 5 APE, and 5 negative-for-PE CTPA exams not included in the test set) to select the optimal epoch from the phase 2 training. We evaluated the local test set using this “locally optimal” model. Apart from the model selection, local data was not used in the model training.

### Data preprocessing

We converted CTPA volumes to NIfTI file format using dcm2niix version 1.0.20201102 [13]. Using MIP requires the removal of the surrounding high-intensity structures, which was achieved by the deep learning-based lung segmentation tool lung mask 0.2.8 by Hofmanninger et al. [14]. This removed the tissues beyond the lungs, *e.g.*, bones, mediastinum, trachea, main bronchi, heart, great vessels, and hila, leaving only the separate left and right lungs with the smaller vessels and bronchi. Masked MIP image appearance was fixed by a radiologist manually choosing optimal colour and opacity transfer functions based on 30 randomly selected CTPA studies of the RSPECT dataset. This adjustment and the following visual inspections were performed on the image processing platform 3D Slicer 4.11 [15]. We considered the values optimal when both the small peripheral and larger proximal vessels delineated well with the least amount of noise from the lung parenchyma in most images.

**Fig. 3:**
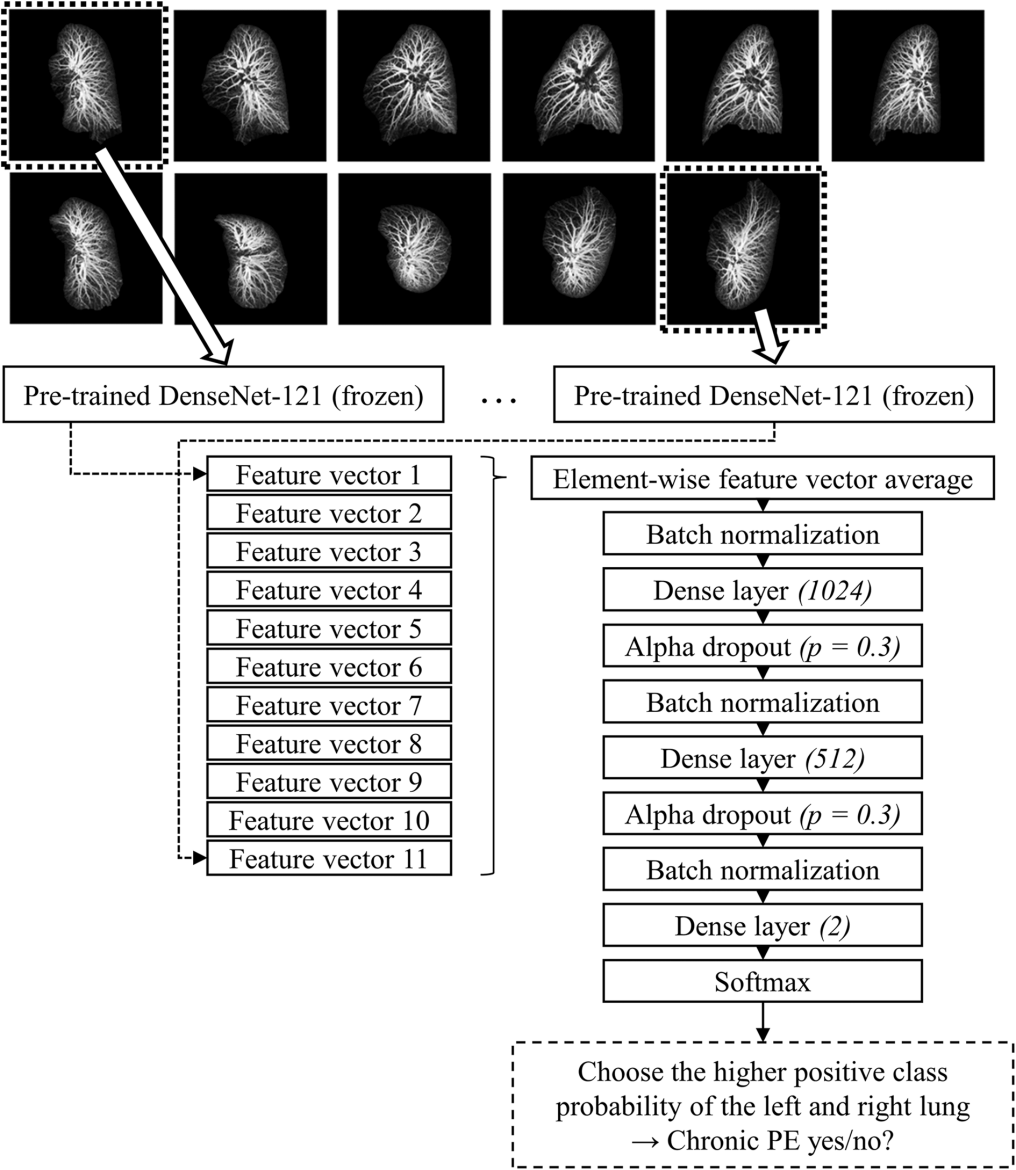
The neural network architecture with DenseNet-121 as the frozen base model. Individual feature vectors were averaged and fed to the three-layer classification network. Lungs were processed separately, and the outputs were either taken individually or combined by taking the maximum of the positive class. Note that only the parameters trained by gradient descent were frozen in the base model, *i.e.*, the batch statistics (the running mean and variance in the batch normalisation layers in the base model) were calculated during fine-tuning. *PE*, Pulmonary embolism

The same transfer functions were used on every masked CTPA volume to generate MIP lung images automatically. MIPs were generated at 11 different angles, rotating the view 150° in 30-degree increments around the vertical and left-to-right-horizontal axis starting from the anteroposterior view (Fig. 3). The MIPs were generated online during CNN training and inference using Python 3.8.8 and Visualization Toolkit 9.0 [16] packaged in the data analysis and visualisation application ParaView 5.9.1 [17].

ParaView headless server distribution provided pre-built libraries allowing easy graphics card utilisation in fast off-screen MIP image rendering. In addition to the typical augmentation by rotating the final projections (hereafter referred to as 2D rotations), the online MIP generation allowed altering the projection direction — three-dimensional (3D) rotations — during training. MIP images were then normalised to 0–1 range. Miscellaneous image processing and analysis tasks (*e.g.*, image sorting, quality checks, and statistical analysis) were performed on MATLAB 2018b (MathWorks, Natick, MA, USA). In the final testing, all processing steps (conversion, segmentation, MIP rendering, and prediction) were run consecutively without a need for user intervention facilitating easy automation.

### Neural network training and visualisation

Model architecture selection and hyperparameter optimisation were performed in phase 1 using fivefold cross-validation with a separate early stopping set. We utilised transfer learning to extract feature vectors, which were fed to a multilayer perceptron classifier outputting probability for CPE. RSPECT dataset labels (diagnoses) were used as the training targets. The chosen general architecture and training parameters were the following. Each of the eleven MIP input images was first separately fed to a frozen “base model” trained on ImageNet [18] with the original classification layer removed. The eleven feature vectors were then averaged and fed to an unfrozen three-layer multilayer perceptron with rectified linear unit activations followed by Alpha Dropout [19] and batch normalisations (Fig. 3).

The network produced predictions for the presence/absence of CPE for both lungs separately. We assume that vascular changes can be present in either or both lungs and therefore use the maximum of the left and right lung CPE prediction (LR_max_) as a more accurate indicator of the disease than individual lung predictions. However, the left and right lungs were used as independent inputs with the same label (CPE or no CPE) during training. Every pair of left and right lungs belonged only to one data set at a time, and the volume order during training was randomised. Model checkpoints were saved after each epoch to facilitate retrospective optimal model selection (*e.g.*, by using local data). We used multiple models to estimate the variation and, more importantly, to compose an ensemble model by averaging the five outputs after softmax activation.

We created localised 3D visualisations for the CPE classification to evaluate the network decision biases, to understand the reasons for possible false-positive and false-negative findings, and to guide the possible future development of the methodology. Instead of a more traditional occlusion approach, we chose an 80 × 80 × 80 mm^3^ patch from the CTPA volume and set all the voxels outside the patch to -1,024 HU. This was then used as an input to the final ensemble model, and the prediction probabilities were recorded. The patch location was then varied until the whole volume was covered. The predictions were saved to the patch locations (taking the maximum of any overlap) providing rough 3D localisation in the same coordinate space as the original CTPA.

As a final network dissection method, we rerun the final ensemble model predictions on the local test set volumes after the intensities below -500 HU had been set to -1,024 HU. This was done to test if parenchymal hypoperfusion, expected to manifest below this threshold, would be a major factor in the CPE detection instead of the assumed vasculature changes.

### Performance metrics

Receiver operating characteristics (ROC) area under the curve (AUC) was used to compare overall model performances agnostic to the operating point selection. Additionally, we used balanced accuracy (BAcc), defined as the average of sensitivity and specificity, instead of normal accuracy to facilitate more straightforward interpretability than AUC and better comparability between sets with varying class balances. Ad hoc balanced accuracy was calculated by choosing the operating point based on the early stopping set and post hoc BAcc by choosing the optimal operating point from the corresponding test set. The prediction threshold that minimises the distance to the top-left corner of the ROC curve was considered optimal. Ad hoc BAcc was defined without prior knowledge of the test set, whereas post hoc BAcc should be regarded only as a summary of the test set.

## Results

### Model selection and performance

According to the cross-validation in experiment A (phase 1), the best-performing base model was DenseNet-121 with random ± 3-degree 2D rotations resulting in 0.70 AUC (Supplemental Table S1). Utilising 3D rotations, a slight 0.02 increase in the average AUC was observed. We used these settings in the rest of the experiments/evaluations. Experiment B, in which the training used more CTPA volumes but without the RV/LV-based exclusion, showed a somewhat poorer performance (0.68 AUC) on the experiment A cross-validation sets. The experiment B cross-validation AUC of 0.60 indicated that no meaningful learning was achieved for this population. This discrepancy may suggest that the possible vasculature changes are more distinct when RV/LV ≥ 1.

Model performance on the stratified RSPECT test sets is summarised in Table 3. Experiment A test AUC was higher than the cross-validation average, potentially indicating that the randomly selected test set might not fully represent the study population. A small difference was observed when using the ensemble model: a slight improvement was seen in the post hoc BAcc from 0.73 to 0.75, and no improvement was seen for the AUC. LR_max_ had poorer performance than treating the lungs individually. We verified that the phase 2 final model did not perform well on test set B (0.63 AUC), *i.e.*, unable to detect cases without the prior RV/LV-based selection.

**Table 3:** Performance metrics of the network trained five times on the experiment A data

Base model	DenseNet-121		
Augmentations	3° rotations in-plane, 10° rotations in 3D		
Set and model	Test set from experiment A Single model (mean ± SD)	Test set from experiment A Ensemble model	Test set from experiment B, Single model (mean ± SD)
AUC, all	0.80 ± 0.02	0.80	0.63 ± 0.01
BAcc (ad hoc), all^a^	0.71 ± 0.02	0.74	0.60 ± 0.01
BAcc (post hoc), all^b^	0.73 ± 0.02	0.75	0.61 ± 0.01
AUC, LR_max_	0.79 ± 0.02	0.80	0.63 ± 0.01
BAcc (ad hoc), LR_max_^a^	0.70 ± 0.02	0.71	0.60 ± 0.01
BAcc (post hoc), LR_max_^b^	0.73 ± 0.01	0.73	0.62 ± 0.02

The operating points for the test set balanced accuracy calculations were chosen by selecting the threshold nearest to the top-left corner of the receiver operator characteristic curve calculated from the early stopping set (^a^) or from the final test set (^b^). *AUC* Area under the receiver operator characteristic curve, *BAcc* Balanced accuracy, *LR*_max_ Maximum of the left and right lung prediction, *SD* Standard deviation

**Fig. 4:**
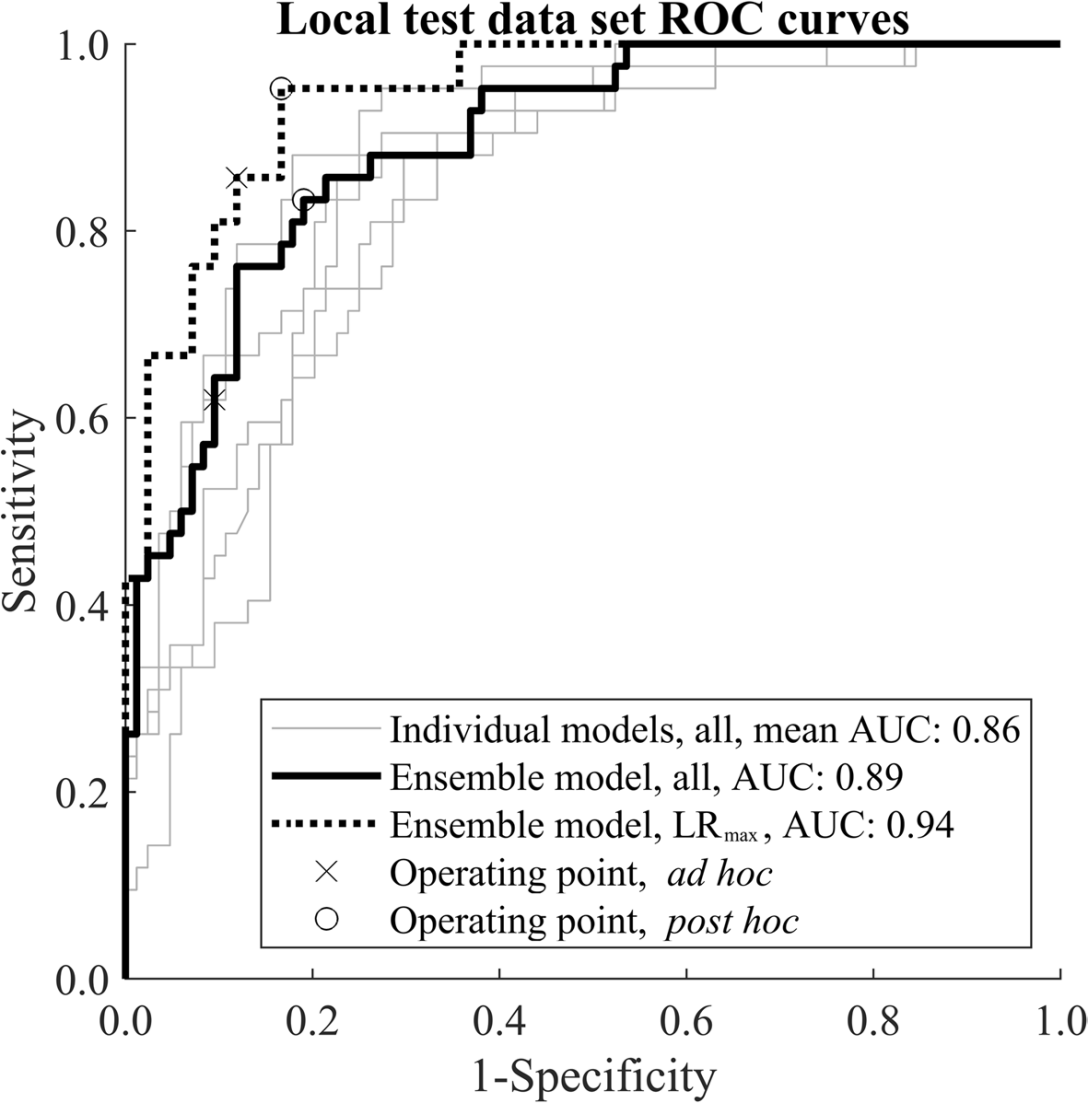
ROC curves of the final individual (light grey) and ensemble models (black). LR_max_ is calculated by taking the maximum of exam’s left and right lung chronic pulmonary embolism predictions, whereas *all* is calculated by treating each lung separately. *AUC*, Area under the curve; *LR*_max_, Maximum of the left and right lung prediction; *ROC*, Receiver operator characteristic

We achieved notably better test performance for the local dataset than the RSPECT set (experiment A column in Table 4), *e.g.*, the average AUC LR_max_ were 0.87 and 0.79, respectively. In phase 3, we used a small local dataset of 15 CTPA volumes for the (“early stopping”) model selection. Using the original training runs was possible because the model checkpoints had been saved for all 30 epochs for all five runs. With a locally optimal ensemble model and considering CPE to be present in either one or both lungs, we achieved a very high AUC of 0.94 and BAcc of 0.89. Although model selection using the tiny local early stopping set outperformed the potentially noisier public dataset, it was found that operating point selection was suboptimal (see ad hoc operating point for the ensemble model for individual lungs (cross for “all” curve) in Fig. 4).

**Table 4:** Performance metrics on the local test data using early stopping from RSPECT (experiment A) or from a small sample of 15 CTPA exams (local data)

Early stopping set	Experiment A early stopping	Local data early stopping	
	Single model (mean ± SD)	Single model (mean ± SD)	Ensemble model
AUC, all	0.82 ± 0.01	0.86 ± 0.04	0.89
BAcc (ad hoc), all^a^	0.69 ± 0.04	0.74 ± 0.07	0.75
BAcc (post hoc), all^b^	0.76 ± 0.02	0.81 ± 0.03	0.82
AUC, LR_max_	0.87 ± 0.01	0.89 ± 0.04	0.94
BAcc (ad hoc), LR_max_^a^	0.67 ± 0.06	0.76 ± 0.07	0.87
BAcc (post hoc), LR_max_^b^	0.82 ± 0.02	0.83 ± 0.05	0.89

The operating points for the test set balanced accuracy calculations were chosen by selecting the threshold nearest to the top-left corner of the receiver operator characteristic curve calculated from the early stopping set (^a^) or from the final test set (^b^). *AUC* Area under the receiver operator characteristic curve, *BAcc* Balanced accuracy, *LR*_max_ Maximum of the left and right lung prediction, *SD* Standard deviation

Using the post hoc operating point in the local test set, all but one CPE were identified correctly. Three patients in the negative exam for the PE group and four in the APE group were misclassified as false negatives. Of the 20 true positives, 19 had RV/LV ≥ 1, and the one false positive had RV/LV ≥ 1. Fifteen of the 35 true negatives and 4 of the 7 false positives had RV/LV ≥ 1.

### Decision visualisations and analysis

In addition to the patient-level performance evaluation, we reviewed the local test set CTPA images, visually analysing the areas where the algorithm showed the highest activation. The network visualisation was based on predicting patches of the original volume as described in “Methods”. For the visual analysis, a probability threshold of 0.80 was selected for the activation maps, which corresponded to the optimal threshold for ROC curves using the maximum of each lung’s patch predictions.

**Fig. 5:**
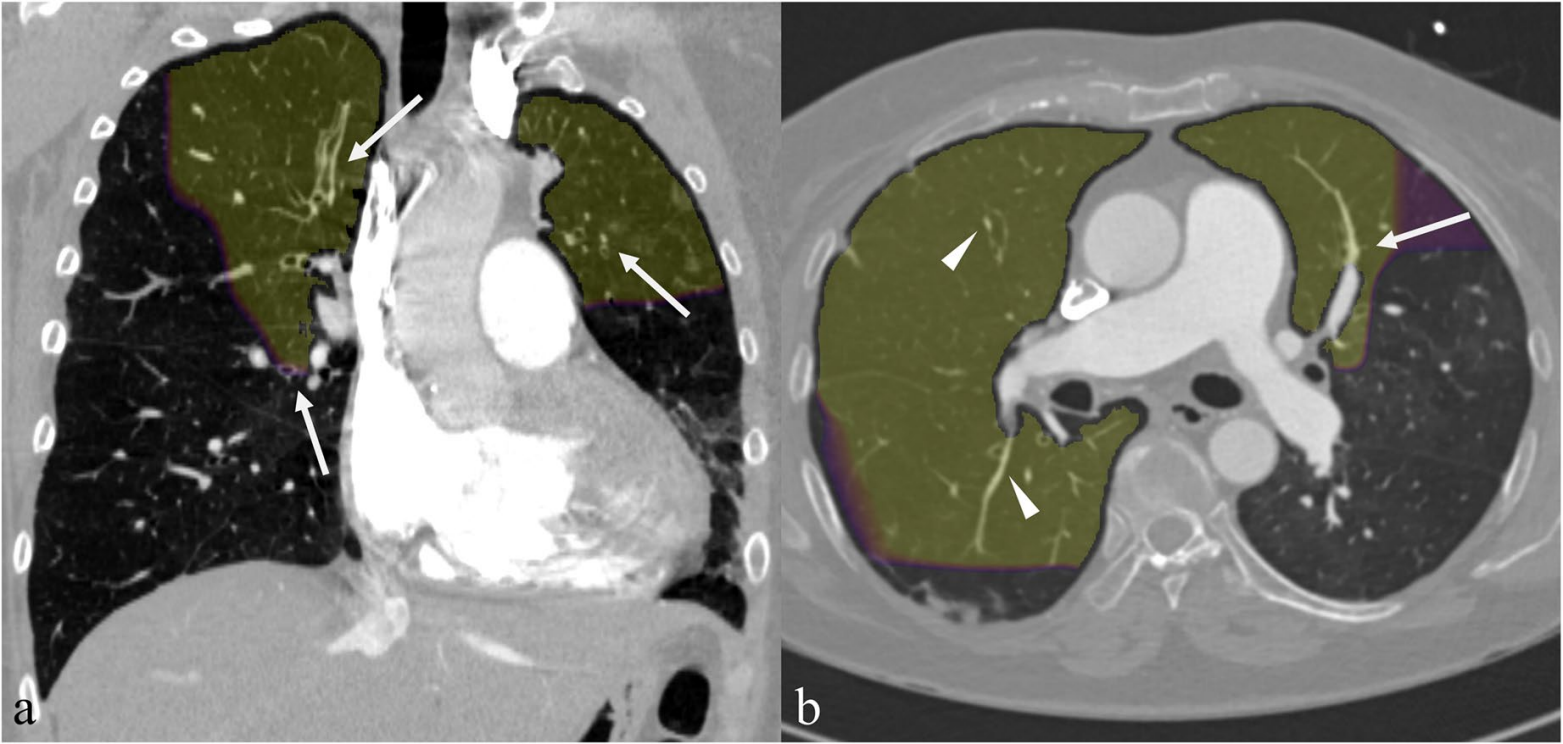
Examples of the CNN prediction visualisations are shown in yellow for two true positive cases. Segmental and subsegmental arteries of the upper lobes are reduced in diameter and are smaller than the accompanying bronchi (arrows in (**a**)). Abrupt narrowing of the left superior lingular artery (arrow in (**b**)) and reduction of pulmonary artery diameter (arrowheads in (**b**)) are seen as signs of chronic pulmonary embolism

**Fig. 6:**
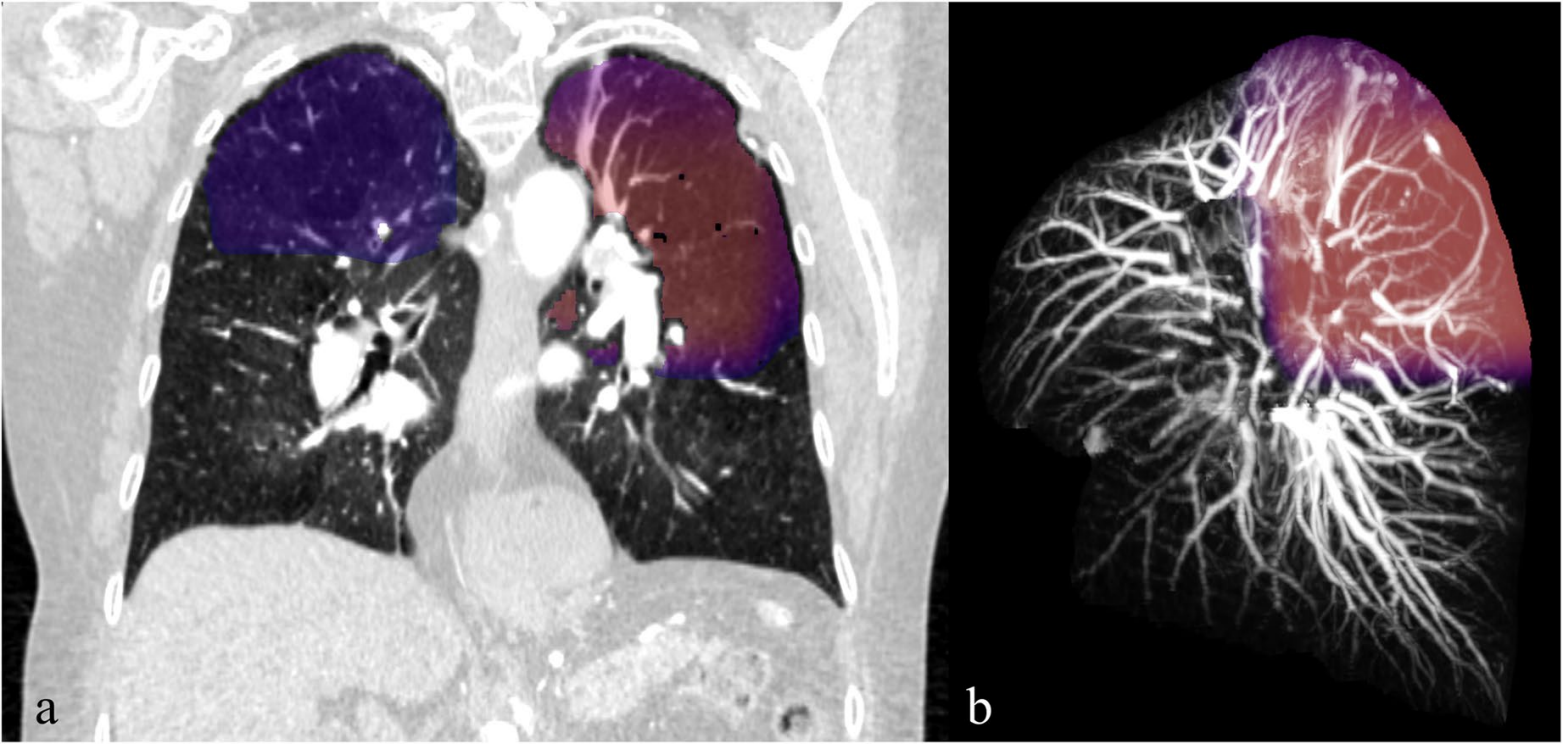
An example of tortuous course vessels in the left lung of a chronic pulmonary embolism patient shown in coronal (**a**) and MIP images (**b**). The right lung is hidden in **b**

In the CPE group, 13 patients had a disparity in vessel size. Twelve patients had abnormally narrow pulmonary segmental or subsegmental vessels in the activation area (see Fig. 5a), and eight patients had a reduction in the number of distal vessels. Six patients had pulmonary artery calibre variation, and four had abrupt narrowing of the vessels (see Fig. 5b). Six patients had tortuous arteries (Fig. 6), and four had dilatation of pulmonary arteries. No apparent CPE-related morphological vascular signs could be found in the activation area in two patients. Two patients had chronic web-like and wall-adherent emboli in the activation area, and one had a lung infarction. In addition, streak artefacts from the contrast material bolus in the upper vena cava were seen in the activation area of two patients.

Five true positives with no activation over the probability threshold of 0.80 in the CNN predictions from the fixed-sized patches were left out of the decision visualisation and analysis. No evident artefacts or other apparent reasons were found in the general analysis of the CTPA of the one false-negative case.

Two false positives had a strong contrast material bolus in the upper vena cava with streak artefacts in the activation areas. One false positive had two proximal acute embolisms, and the distal branches of these arteries were in the activation area. However, there was no embolism in the activation area, and the distal arteries were of normal calibre.

Four false positives did not have any activation over the probability threshold of 0.80 in the CNN predictions from the fixed-sized patches. However, the CTPA images of these patients were analysed in general to evaluate the possible reasons for the false-positive findings.

One of the false positives had a weak concentration of contrast material in the pulmonary arteries, and most of the contrast was already in the systemic arteries. Another false positive had many linear atelectases in the right lower lobe related to unilateral pleural effusion. One had an extensive acute embolism affecting all the segmental branches with perfusion defects in the parenchyma. The last false positive had tortuosity and compression of the pulmonary vessels caused by a mass effect from a sizeable necrotic infiltration relating to a chronic infection in the right middle lobe.

We performed the final analysis on the local test set by first thresholding the CTPAs so that all the voxels with intensities below -500 HU were set to -1,024 HU and effectively masked out. This removed the normal parenchyma beyond the vessels and resulted in 0.87 AUC all and 0.89 AUC LR_max_ using the final ensemble model optimised for the local data. Although a performance hit can be seen, probably because of an altered image appearance or because part of the information on the vessels was also lost, the classification AUC remained high even without retraining the model.

## Discussion

We developed and evaluated a novel CNN algorithm, which analyses the morphology of the pulmonary vasculature in 2D rotational MIP images created from CTPA data and identifies whether the subject has a chronic pulmonary embolism. Although MIP images have been used for CNN training on different modalities in recent studies [20–23], this is the first study on a CNN technique for CPE recognition from MIP images of the pulmonary vasculature. The smallest vessels are better delineated and more accessible to the human eye than in 2D slices. We hypothesised that we could direct the CNN focus on analysing the vessels instead of parenchymal markings or the airways. Also, in the presented model, the feature vectors were combined by element-wise averaging, suggesting a straightforward and efficient way of capturing information from multiple angles (images). MIP was chosen to produce consistent and robust results; although it required extracting the lungs from the CTPA volumes, it did not require the more challenging task of vessel segmentation or other image rendering operations. The algorithm showed a good performance in the classification of CPE with 0.94 AUC and 0.89 BAcc, despite the varied quality of the CTPA in the dataset used for the CNN training.

The AI tool with fast processing could be used as a second reader tool to suggest the presence of chronic pulmonary embolism after the read, as radiologic signs of CTEPH are often missed in CTPA and poorly reported or misclassified as acute PE in daily practice [5, 24]. This might improve the early diagnosis, which is challenging and often delayed, with a median of 14 months after the first symptoms [3]. Further studies are needed to test the CNN performance in clinical settings. Furthermore, by matching CNN findings with specific changes in the vasculature morphology, novel disease features could possibly be identified.

The vascular remodelling and chronic obstruction in CPE may lead to pulmonary hypertension and right heart strain [25]. Since we had no clinical data available in the RSPECT dataset, we predicted that patients with RV/LV ≥ 1 would have more advanced disease and present with more abundant vascular characteristics of CPE. This hypothesis was tested by training the CNN with and without an RV/LV-based exclusion criterion. The first experiment with the RVLV-based exclusion criteria performed better in the public dataset. Although the sample size in the second experiment without the exclusion was almost double in size compared to the first, this may indicate that the CPE vascular characteristics are more distinct in patients with right heart strain.

All but one of the CPE patients were correctly classified by the CNN with the optimal operating point chosen post hoc, with seven false positives in the control groups. One false positive had very weak contrast opacification in the pulmonary arteries, and the correct timing of the contrast injection might be necessary for optimal results in the CNN prediction. Also, streak artefacts relating to the contrast bolus might harm the CNN predictions, as these were seen in two false-positive predictions shading the small vessels in apices of the lungs. In addition, pathological processes, *e.g.*, tumours or infectious masses, which alter the course and calibre of the pulmonary vessels might impair the CNN analysis, as was seen in one false-positive case.

Because of the mosaic perfusion seen in CPE patients, we did an additional test masking the lung parenchyma out of the CTPA, which showed only a slight performance impairment, and the predictive accuracy remained high with an AUC of 0.89. We conclude that low-density parenchymal changes have little effect on the model performance, but the true significance requires further studies.

This study has some limitations. The use of MIP images limits our CNN analysis only to vascular changes, which are shown to differ significantly, at least in CTEPH, compared to controls [26]. However, the radiological diagnosis also depends on other imaging features, such as parenchymal changes or alterations in the heart. Hence, our proposed model might benefit from integrating a CNN algorithm analysing other features in CTPA. Another limitation regarding MIP images is that calcified vessels cannot be accurately evaluated due to the averaging of plaque opacification, and the presence of motion or streak artefacts may mimic abnormalities, which might impair the CNN predictions.

Also, the vessel delineation in MIP images could have been more precise by individually adjusting each study’s settings for the window, level, and opacity values. However, we wanted to create an automatic tool with no human interaction, and the simplest solution for automatic MIP image creation was using universal settings. Full vasculature segmentation could allow exploring 3D rendering methods with our technique.

Our study had a small sample size partly due to the rarity of the CPE [27]. Therefore, we cannot demonstrate the ability of the CNN to distinguish chronic pulmonary embolism from pulmonary hypertension caused by other aetiologies, which might feature similar vascular characteristics.

Also, relating to the small sample size, we only had two CPE cases in our dataset with RV/LV < 1, of which only one had been randomly selected for the test set. Hence, the preliminary results of our study might only be applicable to CPE patients with RV/LV ≥ 1, especially when considering the RV/LV-based exclusion criteria used for the CNN’s training in the public dataset.

Finally, in this retrospective study, we were not able to access all the parameters used for the CT acquisition and contrast media protocols, which may have impacted the image quality and CNN performance. Testing our model on other CPE datasets, CT scanners and facilities are warranted, as well as a further investigation with prospectively acquired images to validate our results.

In conclusion, we developed a novel deep learning model recognising CPE on CTPA images. With excellent predictive accuracy, the proposed model can differentiate chronic pulmonary embolism with RV/LV ≥ 1 from acute pulmonary embolism or non-embolic cases.

## Supplementary Information

**Additional file 1: Supplementary Table S1.** The areas under the receiver operating characteristic curvesfrom the five-fold cross validation runs calculated from the predicted CPE probability maxima of the left and right lungs.

## Data Availability

The public datasets analysed during the current study are available in the RSNA repository, https://www.rsna.org/education/ai-resources-and-training/ai-image-challenge/rsna-pe-detection-challenge-2020 [12]. The trained deep learning models are available from the corresponding author on a reasonable request.

## Abbreviations

2D: Two-dimensional
3D: Three-dimensional
APE: Acute pulmonary embolism
AUC: Area under the curve
BAcc: Balanced accuracy
CNN: Convolutional neural network
CPE: Chronic pulmonary embolism
CT: Computed tomography
CTEPH: Chronic thromboembolic pulmonary hypertension
CTPA: Computed tomography pulmonary angiogram
LR_max_: Maximum of the left and right lung CPE prediction
MIP: Maximum intensity projection
PE: Pulmonary embolism
ROC: Receiver operating characteristics
RSNA: The Radiological Society of North America
RSPECT: The RSNA Pulmonary Embolism CT dataset
RV/LV: Right ventricular to left ventricular diameter ratio
V/Q: Ventilation-perfusion lung scan using scintigraphy

## References

[1] Galiè N, Humbert M, Vachiery J (2015). 2015 ESC/ERS guidelines for the diagnosis and treatment of pulmonary hypertension. Eur Respir J.

[2] Delcroix M, Lang I, Pepke-Zaba J (2016). Long-term outcome of patients with chronic thromboembolic pulmonary hypertension: results from an international prospective registry. Circulation.

[3] Pepke-Zaba J, Delcroix M, Lang I (2011). Chronic thromboembolic pulmonary hypertension (CTEPH): results from an international prospective registry. Circulation.

[4] Klok FA, Barco S, Konstantinides SV (2018). Determinants of diagnostic delay in chronic thromboembolic pulmonary hypertension: results from the European CTEPH registry. Eur Respir J.

[5] Rogberg AN, Gopalan D, Westerlund E, Lindholm P (2019). Do radiologists detect chronic thromboembolic disease on computed tomography?. Acta Radiol.

[6] Ruggiero A, Screaton NJ (2017). Imaging of acute and chronic thromboembolic disease: state of the art. Clin Radiol.

[7] Gopalan D, Blanchard D, Auger WR (2016). Diagnostic evaluation of chronic thromboembolic pulmonary hypertension. Ann Am Thorac Soc.

[8] Bergin CJ, Rios G, King MA, Belezzuoli E, Luna J, Auger WR (1996). Accuracy of high-resolution CT in identifying chronic pulmonary thromboembolic disease. AJR Am J Roentgenol.

[9] Renapurkar RD, Shrikanthan S, Heresi GA, Lau CT, Gopalan D (2017). Imaging in chronic thromboembolic pulmonary hypertension. J Thorac Imaging.

[10] Castañer E, Gallardo X, Ballesteros E (2009). CT diagnosis of chronic pulmonary thromboembolism. Radiographics.

[11] Vainio T, Mäkelä T, Savolainen S, Kangasniemi M (2021). Performance of a 3D convolutional neural network in the detection of hypoperfusion at CT pulmonary angiography in patients with chronic pulmonary embolism: a feasibility study. Eur Radiol Exp.

[12] Colak E, Kitamura FC, Hobbs SB (2021). The RSNA pulmonary embolism CT dataset. Radiol Artif Intell.

[13] Li X, Morgan PS, Ashburner J, Smith J, Rorden C (2016). The first step for neuroimaging data analysis: DICOM to NIfTI conversion. J Neurosci Methods.

[14] Hofmanninger J, Prayer F, Pan J (2020). Automatic lung segmentation in routine imaging is primarily a data diversity problem, not a methodology problem. Eur Radiol Exp.

[15] Kikinis R, Pieper SD, Vosburgh KG, Jolesz F (2014). 3D Slicer: a platform for subject-specific image analysis, visualization, and clinical support. Intraoperative imaging and image-guided therapy.

[16] Schroeder W, Martin K, Lorensen B (2006). The visualization toolkit: an object-oriented approach to 3D graphics.

[17] Ayachit U (2015). The ParaView guide: a parallel visualization application.

[18] Deng J, Dong W, Socher R, Li LJ, Li K, Fei-Fei L (2009) ImageNet: a large-scale hierarchical image database. In: 2009 IEEE Conference on computer vision and pattern recognition, Miami, 20–25 June 2009, pp. 248–255. 10.1109/CVPR.2009.5206848

[19] Klambauer G, Unterthiner T, Mayr A, Hochreiter S (2017) Self-normalizing neural networks. In: Guyon I, Von Luxburg U, Bengio S, et al (Eds) Advances in neural information processing systems 30, Long Beach, 2017.

[20] Nakao T, Hanaoka S, Nomura Y (2018). Deep neural network-based computer-assisted detection of cerebral aneurysms in MR angiography. J Magn Reson Imaging.

[21] Zheng S, Cui X, Vonder M (2020). Deep learning-based pulmonary nodule detection: Effect of slab thickness in maximum intensity projections at the nodule candidate detection stage. Comput Methods Programs Biomed.

[22] Fujioka T, Yashima Y, Oyama J (2021). Deep-learning approach with convolutional neural network for classification of maximum intensity projections of dynamic contrast-enhanced breast magnetic resonance imaging. Magn Reson Imaging.

[23] Takahashi K, Fujioka T, Oyama J (2022). Deep learning using multiple degrees of maximum-intensity projection for PET/CT image classification in breast cancer. Tomography.

[24] Klok FA, Delcroix M, Bogaard HJ (2018). Chronic thromboembolic pulmonary hypertension from the perspective of patients with pulmonary embolism. J Thromb Haemost.

[25] Hoeper MM, Mayer E, Simonneau G, Rubin LJ (2006). Chronic thromboembolic pulmonary hypertension. Circulation.

[26] Rahaghi FN, Ross JC, Agarwal M (2016). Pulmonary vascular morphology as an imaging biomarker in chronic thromboembolic pulmonary hypertension. Pulmon Circ.

[27] Delcroix M, Kerr K, Fedullo P (2016). Chronic thromboembolic pulmonary hypertension. Epidemiology and risk factors. Ann Am Thorac Soc.

