# Supplementary material for: Leveraging open dataset and transfer learning for accurate recognition of chronic pulmonary embolism from CT angiogram maximum intensity projection images
Source: Eur Radiol Exp. 2023 Jun 21;7:33. doi: 10.1186/s41747-023-00346-9 (PMC10281920; doi:10.1186/s41747-023-00346-9)
Supplement: Supplementary file 1 — Additional file 1: Supplementary Table S1. The areas under the receiver operating characteristic curvesfrom the five-fold cross validation runs calculated from the predicted CPE probability maxima of the left and right lungs. [file 41747_2023_346_MOESM1_ESM.pdf]

Leveraging open dataset and transfer learning for accurate recognition of chronic pulmonary embolism from CT angiogram maximum intensity projection images

Convolutional neural network training details

The network implementation and gradient descent optimisation were done using PyTorch 1.11 [1], cross-entropy loss function, Adam optimiser with learning rate 10^{-4}, weight decay 10^{-4}, and batch size 16. The best model was chosen when the maximum of the left and right lung (LR_{max}) area under the receiver operating characteristic curve (ROC AUC) stopped improving in the early stopping set.

Experimental results using the training parameters above are reported in Supplementary Table S1 for the following pre-trained base models for experiment A: DenseNet-121 [2], RegNetX-32GF [3], ResNet-152 [4], ResNeXt-101 [5], SqueezeNet1_1 [6], and VGG19 [7]. Different levels of 3D rotation augmentation were tested on the best-performing model (±3-degree and ±10-degree are reported). The best-performing model and augmentation approach were used in experiment B and in training the final models. The final model was trained on a remote cluster five times for 30 epochs, after which they were transferred to a local environment for local data set model selection and analysis. An ensemble model was created by averaging the five outputs after softmax activation.

The amount of data was drastically reduced using 2D MIP images instead of all the CTPA slices. The total time for loading the model, the automatic MIP reconstructions, and CNN inference take approximately only five seconds for a single study on NVIDIA Quadro M6000.

References

[1] Paszke A, Gross S, Massa F, et al (2019) Pytorch: An imperative style, high-performance deep learning library. In: Wallach HM, Larochelle H, Beygelzimer A, d'Alché-Buc F, Fox EA, Garnett R (Eds) Advances in neural information processing systems 32, Vancouver, 2019.

[2] Huang G, Liu Z, Van Der Maaten L, Weinberger KQ (2017) Densely connected convolutional networks. In: 2017 IEEE Conference on computer vision and pattern recognition (CVPR), Honolulu, 21–26 July 2017, pp. 2261–2269. https://doi.org/10.1109/CVPR.2017.243

[3] Radosavovic I, Kosaraju RP, Girshick R, He K, Dollár P (2020) Designing network design spaces. In: 2020 IEEE/CVF Conference on computer vision and pattern recognition (CVPR), Seattle, 13–19 June 2020, pp. 10425–10433. https://doi.org/10.1109/CVPR42600.2020.01044

[4] He K, Zhang X, Ren S, Sun J (2016) Deep residual learning for image recognition. In: 2016 IEEE Conference on computer vision and pattern recognition (CVPR), Las Vegas, 27–30 June 2016, pp. 770–778. https://doi.org/10.1109/CVPR.2016.90

[5] Xie S, Girshick R, Dollár P, Tu Z, He K (2017) Aggregated residual transformations for deep neural networks. In: 2017 IEEE Conference on computer vision and pattern recognition (CVPR), Honolulu, 21–26 2017, pp. 5987–5995. https://doi.org/10.1109/CVPR.2017.634

[6] Iandola FN, Han S, Moskewicz MW, Ashraf K, Dally WJ, Keutzer K (2016) SqueezeNet: AlexNet-level accuracy with 50x fewer parameters and <0.5MB model size. arXiv:1602.07360. https://doi.org/10.48550/arXiv.1602.07360

[7] Simonyan K, Zisserman A (2014) Very Deep Convolutional Networks for Large-Scale Image Recognition. arXiv:1409.1556. https://doi.org/10.48550/arXiv.1409.1556

Supplementary Table S1. The areas under the receiver operating characteristic curves (AUC) from the five-fold cross validation runs calculated from the predicted CPE probability maxima of the left and right lungs (LR_{max}).

Base model	*Train set*	*Validation set*	*AUC, LR_{max} mean (range)*
**DenseNet-121***	**A**	**A**	**0.70 (0.65–0.76)**
RegNetX-32GF	A	A	0.66 (0.50–0.75)
ResNet-152	A	A	0.68 (0.64–0.72)
ResNeXt-101	A	A	0.70 (0.66–0.72)
SqueezeNet1_1	A	A	0.65 (0.54–0.74)
VGG19	A	A	0.68 (0.60–0.75)
DenseNet-121, 3° rotations in 3D	A	A	0.70 (0.64–0.78)
**DenseNet-121, 10° rotations in 3D**	**A**	**A**	**0.72 (0.67–0.80)**
DenseNet-121, 10° rotations in 3D	B	A	0.68 (0.65–0.70)
DenseNet-121, 10° rotations in 3D	B	B	0.60 (0.56–0.65)

* The best performing model, DenseNet-121 (with marginally higher AUC 0.702 than ResNeXt-101 AUC 0.697) was chosen for further experimentation and for experiment B. Three-degree 2D rotation was used as augmentation in all the experiments. No 3D rotation was used in the initial base model selection. *AUC*, area under the receiver operator characteristic curve; *LR_{max}*, maximum of the left and right lung prediction.
